# 

**Published:** 1883-02

**Authors:** 


### CONTENTS:

ART. I.—Bromide of Ethyl, the Most Perfect Anæsthetic for Short, Painful Surgical Operations. By Prof. Julian J. Chisolm, M. D. .......... 433

ART. II.—The Etiology of Dental Decay (Continued). By Geo. Watt, M. D., D. D. S. .......... 446

ART. III.—Cells and Their Relation to Organisms. By Geo. A. Piersol, M. D. .......... 462

Alabama State Dental Association .......... 471

### EDITORIAL, ETC.

Vaccine Virus .......... 473

Dr. Marshall H. Webb .......... 474

Valuable Contributions .......... 475

Diagram of an Incisor Tooth .......... 476

A New Discovery .......... 477

Annual Commencement of the Dental Department of the University of Maryland .. 477

### OBITUARY, ETC.

William Fiero Eddington, M. D., D. D. S. .......... 477

### MONTHLY SUMMARY.

Professional Courtesy .......... 478

Malformation of the Jaws of a Horse .......... 479

To Disguise the Odor of Iodiform .......... 479

Decolorization of the Hair from Neuralgia .......... 480

Excision of the Tongue .......... 480

